# Close orthopedic surgery skin incision with combination of barbed sutures and running subcuticular suturing technique for less dermal tension concentration: a finite element analysis

**DOI:** 10.1186/s13018-023-03755-z

**Published:** 2023-05-05

**Authors:** Li Li, Qin Shao, Wenbin He, Tao Wang, Fang Wang

**Affiliations:** Department of Orthopaedics and Traumatology, Shanghai East Hospital, School of Medicine, Tongji University, Shanghai, 200120 China

**Keywords:** Suture technique, Finite element method, Barbed sutures

## Abstract

**Background:**

Mechanical forces have an important role in the initiation and progression of orthopedic surgical incisions complications. To avoid incision complications with the reduction of dermal tension, surgeons may choose a buried continuous suture technique other than the traditional interrupted vertical mattress suture. Absorbable barbed sutures are widely used in orthopedics due to their convenience and reducing wound tension. The aim of this research is to compare and explain the advantages of running subcuticular suturing technique with absorbable barbed sutures for orthopedic surgical incisions closure.

**Methods:**

Finite element models of layered skin and two different suture techniques, running subcuticular suture and intradermal buried vertical mattress suture, ware constructed. The mechanical property difference between standard sutures and barbed sutures was modelled using different contact friction coefficient. Pulling the skin wound was simulated, and the sutures’ pressure on the skin tissue was determined.

**Results:**

Compared with traditional smooth sutures, the barbed sutures effectively increased the contact force for subepidermal layers, which led the less force variation between different layers. The results also suggested that subcuticular suture caused less stress concentration compared with intradermal buried vertical mattress suture.

**Conclusions:**

In conclusion, our study indicated that running subcuticular suturing technique with absorbable barbed sutures for orthopedic surgical incisions closure results in more uniform stress distribution in the dermis. We recommend this combination as the preferred method of skin closure in orthopedic surgery unless contraindicated.

## Introduction

Keloid and hypertrophic scars (HSs) are common complications of orthopedic surgical incisions. They occur frequently at particular sites, such as the scapular area and the suprapubic region [1]. This is due to the pressure on the skin when the suture is left in place, which can be aggravated by tension on the wound, large bites of tissue, edema, and infection [2]. These conditions are particularly common in patients with extremity fractures, where soft tissue edema results in high tension and long duration of the surgical incision. A murine model of hypertrophic scarring suggested that mechanical forces may be primarily responsible for such scars’ generation in wounded skin, not only strongly promotes their growth [3]. The finite element method (FEM) also clarified that stretching tension is an important condition associated with keloid growth [4].

To avoid incision complications or “railroad tracks” with the reduction of dermal tension, surgeons may choose a buried suture technique other than the traditional interrupted vertical mattress suture. The usage of the buried continuous suture techniques has been prompted by the unpleasant appearance of visible scars on the limbs, the cost of stitch removal and dressing change care, and the inconvenience of traveling to and from the hospital for the elderly. Running subcuticular suture and intradermal buried vertical mattress suture are two common buried suture techniques invented to close wounds with reduced surgical scars [2, 5]. These two suture techniques avoid the uneven distribution of tension throughout the wound associated with surgical knots, and the higher tension burden placed at the knots. It remains unclear which one of these two suturing techniques applied to orthopedic wounds has the better biomechanical advantage in reducing skin tension.

Some other efforts to reduce scarring are improved sutures. Given the excessive relative wound tension and the reasonable concerns of surgeons for suture failure due to tissue-suture slippage using smooth sutures, there is a natural tendency toward overcoming these concerns by over-tightening every stitch in running suture. Excessive pressure within this tissue can produce enzymatic degradation and result in a loss of wound strength and a higher incidence of wound dehiscence [6, 7]. An inevitable partial stitch removal due to infection or “cheese-wiring” can cause the complete strength loss throughout the wound due to the breakage of smooth sutures, which high risky leading entire wound to disintegrate. Barbed suture technology was developed to allow wound closure using a self-anchoring suture that avoids the requirement for knot tying and the loops they entail. In orthopedic surgeries, absorbable barbed sutures are widely used due to their convenience and reducing wound tension [8]. Different from smooth sutures, these barbs aid to maintain tensile strength by continuously gripping the sutured tissue [9]. However, the mechanical interaction between the barbed suture and the tissue as well as the biomechanical advantages compared to conventional smooth sutures still remain unclear.

A few 2D and 3D finite element (FE) models have been developed to study the biomechanics of wound closure and skin tension [4, 10, 11]. It is more reasonable to simulate the interaction between the skin tissue and the sutures with 3D finite element models, as the sutures travel through various skin tissue layers, with each layer varying greatly in mechanical parameters.

Therefore, the aim of this research is to compare and evaluate the biomechanical properties for surgical wound dehiscence using numerical simulation. In the FE simulation, four realistic 3D computational wound models (two different suturing techniques with two different sutures) were built to (a) evaluate the biomechanical differences between intradermal buried vertical mattress suture and running subcuticular suture, (b) compare tissue stress reduction on the tissues’ contact surfaces to the sutures with barbed sutures.

## Methods

### Geometrical modeling

**Fig. 1:**
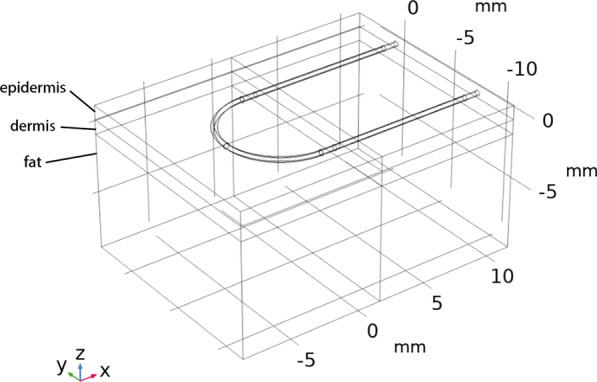
The geometry model of skin with three layers and a single stitch of running subcuticular suture

**Fig. 2:**
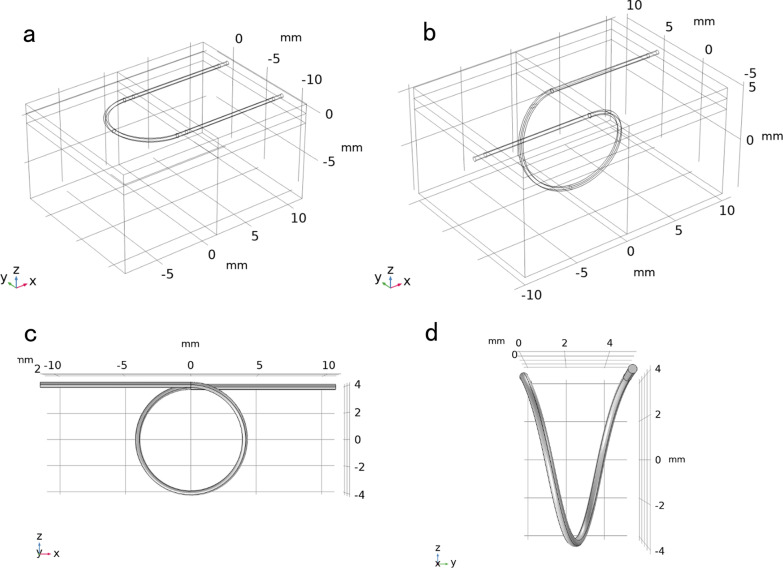
The geometry models of two different suture techniques. **a** A single stitch of running subcuticular suture. **b** A single stitch of running intradermal buried vertical mattress suture. **c** The frontal view of the intradermal buried vertical mattress suture model. **d** The left side view of the intradermal buried vertical mattress suture model

As the aim of the FE model was to evaluate the biomechanical properties, including wound tension and dehiscence characters that related to sutures and suturing techniques, the geometry should be able to reflect the necessary and general suture techniques for surgical wound closure with sufficient mechanical information in details. The skin wound included two symmetrical layered skin sections. Each skin section consisted of three layers, including epidermis, dermis and fat with the thickness of 0.98 mm, 1.0 mm and 8.9 mm, respectively [10–13]. The single section of the skin tissue was modelled in the size of 15 mm × 10 mm × 10 mm. A single stitch of running subcuticular suture with skin was modelled to represent a generalized section of a wound, so that the computational resources can be mostly utilized to refine the mesh element to study the interaction between the sutures and the skin tissue (Fig. 1). The suture diameters were set to 0.2 mm, which is the ideal size of USP 3–0 suture [9]. Two different suture techniques were modelled as shown in Fig. 2. The techniques of intradermal buried vertical mattress suture and running subcuticular suture were idealized with constant radius curvature with R = 4 mm. The single stitch of subcuticular suture was idealized in a U-shaped tunnel in the dermis. The single stitch of intradermal buried vertical mattress suture was modelled in a spiral tunnel. In both suture techniques, the sutures did not pass through epidermises [2, 5].

### FE modeling

#### Meshing

**Fig. 3:**
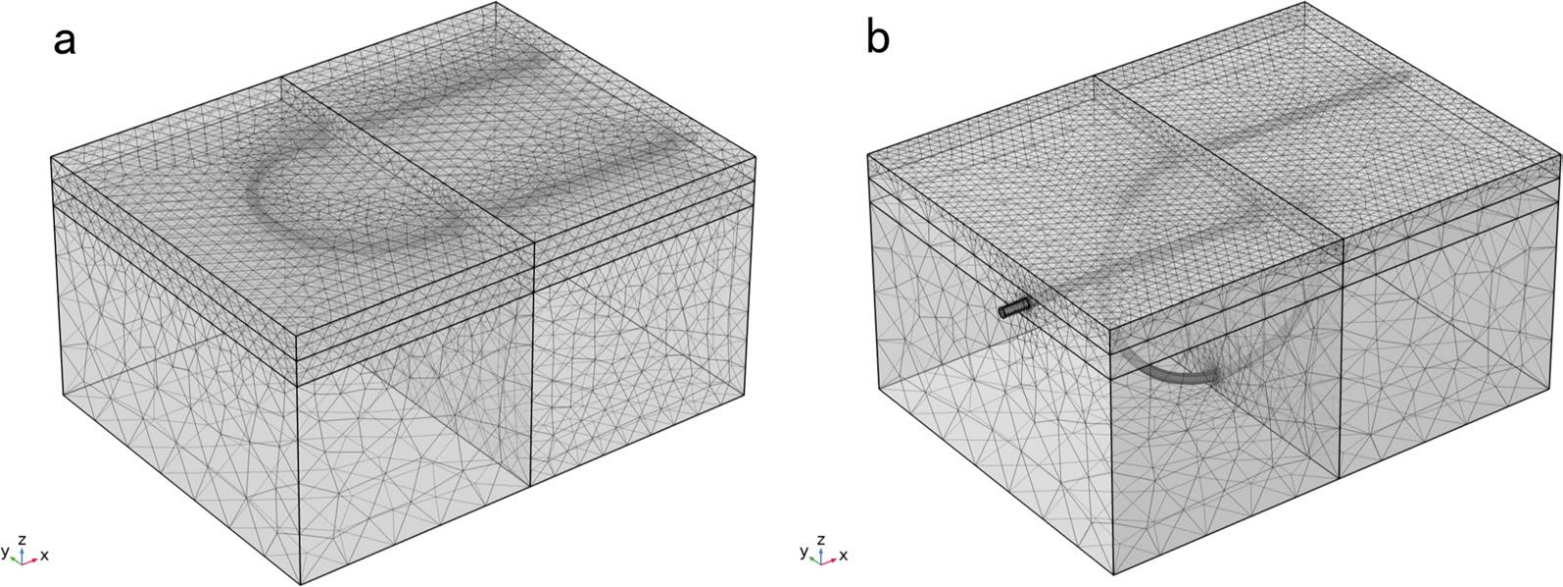
The meshes of two suture techniques. **a** The meshes of the one single stitch of running subcuticular suture mode. **b** The meshes of the one single stitch of intradermal buried vertical mattress suture model

The mesh of the subcuticular suture model contained 445,036 elements with the minimal element size of 0.03 mm (Fig. 3a). The geometry of the intradermal buried vertical mattress suture model was more complicated than that of the subcuticular suture model as more elements were required for the spiral shaped sutures. It contained 673,313 elements with the minimal element size of 0.03 mm (Fig. 3b).

#### Loads and boundary conditions

The mechanical deformation and contact models were implemented for skin suture modelling. As the aim of this study is to compare and evaluate the tissue tension difference between the suturing techniques and sutures at relatively small deformation, it is reasonable to use the linear elastic material model for skin and suture models. The suture process was carried out at a steady and slow motion. The dynamic forces were neglected. The deformation equation of the materials can be expressed by

1 $$\nabla \cdot \left( {{\text{FS}}} \right)^{{\text{T}}} + {\mathbf{F}}_{{\text{V}}} = 0$$

where ${\mathbf{F}} = {\mathbf{I}} + \nabla {\mathbf{u}}$ is the deformation tensor, **u** is the displacement vector, **I** is the identity tensor, **S** is the second Piola–Kirchhoff stress tensor and **F**_**v**_ is the body load. The constitutive equation can be expressed as

2 $${\text{S}} = {\text{JF}}^{ - 1} \left( {{\mathbf{C}}:\varepsilon } \right){\text{F}}^{{ - {\text{T}}}}$$

where **J** is the volumetric deformation, **C** is the material constitutive tensor and **ε** is the strain tensor. The stress can be calculated by

3 $$\sigma_{ij} = C_{ijkl} \varepsilon_{kl}$$

The interface between sutures and skin tissues was modelled by contacting models. The mechanical property difference between standard sutures and barbed sutures was modelled using different contact friction coefficient. The friction coefficients of the standard sutures and barbed sutures were zero and 0.3, respectively [14]. The contact pressure **T**_**n**_ was calculated using the penalty factor and contact gap by

4 $$T_{n} = - p_{n} g_{n} + p_{0}\,if\,g_{n} < p_{0} /p_{n}$$

where **p**_**n**_ is the penalty factor, **g**_**n**_ is the gap and **p**_**0**_ is the pressure at zero gap. The penalty factor can be interpreted as the stiffness of a spring inserted between the contact surfaces. Certain degree of overlapping could occur while **p**_**0**_ was zero.

**Fig. 4:**
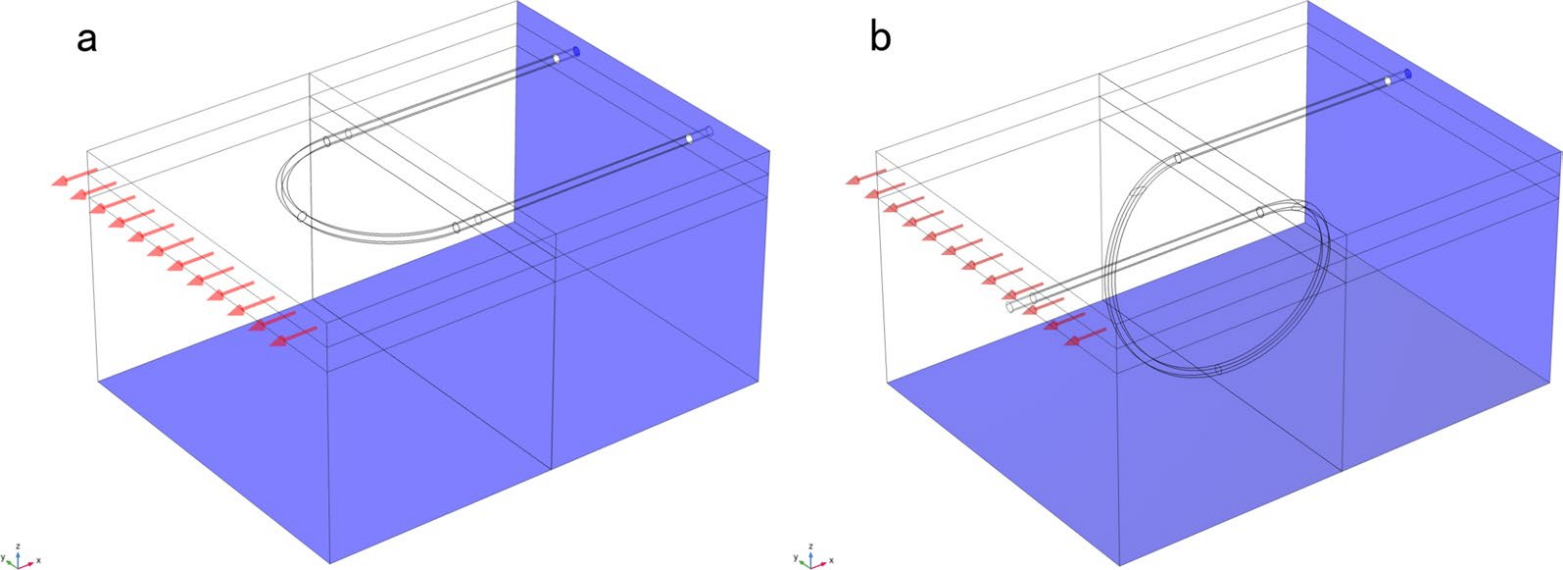
The loads and boundary conditions. **a** A displacement was applied to the epidermis layer to simulate the skin stretch and both ends of the suture were fixed. **b** The left end of the suture was pulled with the epidermis and the right end of the suture was fixed

In this study, the initial pressure between the skin tissue and the sutures was estimated based on the assumption that the sutures are able to expand the suture tunnels to the suture diameters. The simplified FE models were established to model a single stitch passing the tissue. The suture pressure was applied to the cross section of 0.2 mm width, which represents the USP 3–0 suture. The displacement was calculated under different pressure. The pressure that generated the 0.1 mm displacement was 100 kPa, which was considered to be the suture initial contact pressure at zero gap. The prescribed displacement was applied to the epidermis layer to simulate the skin stretch (Fig. 4).

#### Material modeling

The soft tissues are close to volumetrically incompressible [15]. The Poisson’s ratios were close to 0.5 for the tissues. The material properties are listed in Table 1.

**Table 1:** The material properties of the FE model

Material	Elastic modulus (MPa)	Poisson’s ratio
Epidermis [10]	5.000	0.49
Dermis [16]	0.110	0.49
Fat [15]	0.008	0.49
Sutures [10]	130.000	0.20

## Results

**Fig. 5:**
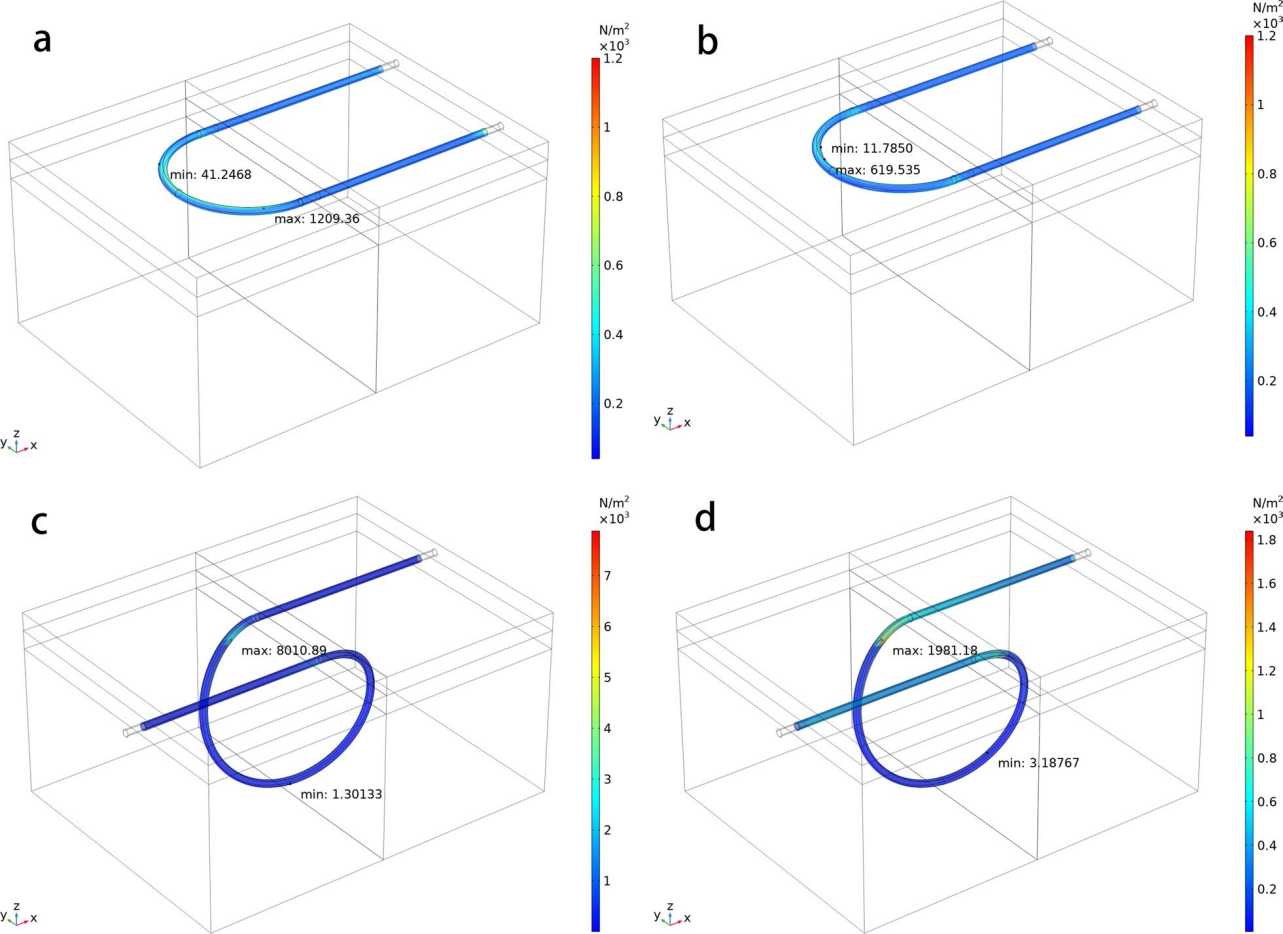
The stress distribution on the tissues’ contact surfaces to the sutures. **a** Combination of running subcuticular suturing technique and smooth suture. **b** Combination of running subcuticular suturing technique and barbed suture. **c** Combination of intradermal buried vertical mattress suturing technique and smooth suture. **d** Combination of intradermal buried vertical mattress suturing technique and barbed suture

Four suture models were developed with two different suture techniques and two different surgical sutures. The stresses of the tissues under stretch loads were simulated as shown in Fig. 5. For the subcuticular suture with smooth suture the maximum stress reached 1.21 kPa under 0.04 mm stretch, which located near the bending tunnel. For the intradermal buried vertical mattress suture the maximum stress reached 8.01 kPa under 0.04 mm stretch. The reason of the difference was that the contact area difference at dermis. As dermis has higher elastic modulus and strength, it was able to undertake more load at same deformation. For subcuticular suture technique, all the suture pass was in the dermis layers. For the intradermal buried vertical mattress suture technique, only partial of the suture pass through dermis layers. The results suggested that subcuticular sutures caused less stress concentration compared with intradermal buried vertical mattress.

The maximum stress of the tissues using barbed suture was 0.62 kPa for subcuticular suture and 1.98 kPa for intradermal buried vertical mattress suture. The proportion of the stress reduction was more significant for subcuticular suture compared with for intradermal suture. The reason was that the stress concentration for intradermal buried vertical mattress was due to the limited contact area in dermis layers. The barbed sutures effectively increased the contact force for subepidermal layers, which led the less force variation between different layers. Regardless the proportional difference for intradermal buried vertical mattress, subcuticular sutures caused less stress concentration using both standard sutures and barbed sutures.

## Discussion

Suturing practices vary widely depending on the surgeon and type of wound operated. Incorrect suture technique and material selection, as well as the nature of the wound, can lead to surgical incision complications such as cheese-wiring, infection, necrosis, dehiscence, and keloids. Mechanical forces have an important role in the initiation and progression of these complications [17]. Tissue edema and the volume of the implant cause considerable dermal tension when the skin incision is closed in orthopedic surgery. This tension was increased by joint movement during early postoperative rehabilitation exercises.

When sutures are put in an interrupted pattern, the whole wound strain is directed to a few important closure spots [8]. Individual suture loops are subjected to excessive tension, which might result in localized ischemia [17, 18]. Microangiographic investigations have shown that wound edges closed with tight suture loops have limited blood flow, resulting in tissue necrosis [19]. The main causes of wound dehiscence include pressure-induced ischemia and necrosis, which predispose the wound to infection [20].

The tension concentration that happens with interrupted sutures is avoided with continuous sutures. The greater friction between the barbed sutures and the tissue helps surgeons overcome the impulse of over-tightening every stitch in continuous sutures. The axially placed escarpments spaced along the length of the suture allow diffuse distribution of tensions along the entire length of the wound, which may provide additional benefits with respect to safety and cosmesis. As indicated in Table 2, whether employing running subcuticular suture or intradermal buried vertical mattress suture, barbed sutures stretched the dermis more equally than smooth sutures.

**Table 2:** The maximum and minimum stress on the dermis from the sutures (Pa)

Suture type	Running subcuticular suture		Intradermal buried vertical mattress suture	
	Minimum	Maximum	Minimum	Maximum
Smooth suture	41.25	1209.36	1.30	8010.89
Barbed suture	11.79	619.54	3.19	1981.18

Wounds subject to excessive tension can also result in wider and more unsightly scars [24]. Intrinsic mechanical forces, including as tension, shear force, osmotic pressure, and hydrostatic pressure, are vital in cutaneous wound healing, with cells converting mechanical inputs into electrical or chemical signals [21]. Natural mechanical qualities vary by body location and have been recognized as key etiological variables in keloid development, with areas of more mechanical stimulation having a higher keloid incidence [22, 23]. Keloids develop and spread both vertically and horizontally, with the direction of their horizontal growth resulting in distinct morphologies that vary depending on where they are. The keloid growth patterns reflect the primary directions of skin tension, which is corroborated by finite element analysis [4]. Skin tension manipulation has been shown to be useful in the prevention and treatment of scars in clinical applications. Because of the foregoing data and how keloids and HTS originate in the dermis, it is hypothesized that reducing the stress on the wound dermis might minimize the chance of postsurgery keloid and HTS development [24, 25]. As a result, Hans employed subcutaneous/fascial tensile reduction sutures, which put the stress on the deep fascia and superficial fascia layers, allowing the wound edges to be connected spontaneously under very tiny tension without the need of dermal sutures [24, 26]. According to early research, this method may help to prevent the formation of big scars [24, 25].

The stress on the dermis in the horizontal direction from the sutures is the crucial signal since the key to preventing scar creation using an optimum suture method is to minimize tension in the dermis. When utilizing the intradermal buried vertical mattress suture, the sutures travel through the fat layer and the dermis, whereas the running subcuticular suture travels entirely through the dermis. Because fat is softer and more sensitive to deformation than the dermis, it is ineffective at balancing tension when the tissue mass is torn apart. Intradermal buried vertical mattress suture has less dermal tissue in contact with the suture, resulting in greater tension.

Scar-less sutures had previously been widely utilized in aesthetic surgery, and as patient demand grows, cosmetic sutures are increasingly being employed in orthopedic surgery. Fully buried sutures are often utilized following thoracolumbar spine and hip surgery with few complications, while interrupted suture is more advised for other locations such as wounds after heel internal fixation. To promote better wound healing, it is necessary to research and select appropriate suturing techniques and tools. Based on our findings, we advocate using barbed sutures in conjunction with running subcuticular suture to close orthopedic surgical skin incisions unless contraindicated.

When compared to the combination of running subcuticular suture and intradermal buried vertical mattress suture, the use of the intradermal buried vertical mattress suture as a single suture in closing the surgical neck incision produced the same suture and better cosmetic effects [5]. It should be observed that the neck skin is lax, and the dermis can be spontaneously aligned with low tension once the subcutaneous tissue is sutured. Dermal sutures are required during orthopedic surgery due to increased pressure in the dermis caused by tissue edema and implant volume.

**Fig. 6:**
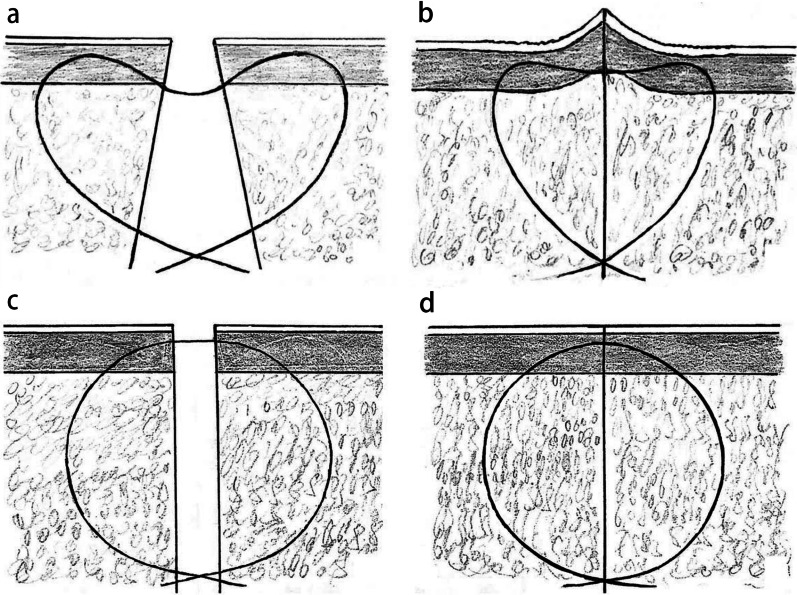
Cross-sectional view depicting the modified buried vertical mattress suture (heart-shaped suture) before (a) and after (b) knotting and the buried intradermal suture before (c) and after (d) knotting [27]

The modified buried vertical mattress suture (heart-shaped suture) is currently commonly used in obstetrics and other professions, and is gaining popularity in orthopedics, but its biomechanical benefits are rarely reported [27]. Because of the larger bite of dermis in a single stitch, its decompression effect should be superior to that of the intradermal buried vertical mattress suture (Fig. 6). With satisfactory clinical response, barbed sutures are frequently utilized in further aspects of orthopedic surgery, such as sealing the joint capsule [28].

It is the first time that the effect of suturing techniques and sutures used to close orthopedic skin incisions has been studied using 3D finite elements method. This experiment is a preliminary exploration, the model only selects one stitch in the suture instead of simulating the whole wound, and does not consider the individualized mechanical characteristics of wounds in various physical regions. Hopefully, it will be the cornerstone of research on wound closure mechanics.

## Conclusions

In conclusion, our study indicated that running subcuticular suturing technique with absorbable barbed sutures for orthopedic surgical incisions closure results in more uniform stress distribution in the dermis. We recommend this combination as the preferred method of skin closure in orthopedic surgery unless contraindicated.

## Data Availability

The datasets used and/or analyzed during the current study are available from the corresponding author on reasonable request.

## Abbreviations

HSs: Hypertrophic scars
FEM: Finite element method
FE: Finite element
